# Hospital Meetings

**Published:** 1896-04-04

**Authors:** 


### HOSPITAL MEETINGS.

### THE ROYAL HOSPITAL FOR CHILDREN AND WOMEN.—ANNUAL MEETING.

The Right Hon. the Lord Mayor presided over the annual meeting of the Royal Hospital for Children and Women, Waterloo Bridge Road, on 20th inst. He said that it was his pleasing duty to propose the adoption of a report which showed that those who compiled it knew well how to lay facts before the public. The financial condition of the hospital was fairly satisfactory, for although no institutions were overburdened with funds, a sufficiency might well be hoped for in this case, when the demand was on so moderate a scale. Emphasising the claims of the sick and poor, the Lord Mayor referred to Carlyle's statement, that if the poor be neglected they have a nasty way of turning upon those who ignore them; they breed diseases, which spread to all classes, and "we pay for our neglect both in health and pocket." This fact should, the Lord Mayor thought, make everyone consider the interests of the hospitals, from selfish and personal as well as philanthropic reasons. Mr. Lawrence spoke with pride of the hospital as a City institution. It relied on the City for its support; the district being so poor and so densely populated that it really demanded more help than the East-end, of which so much more was known. The interest taken in the patients by the lady visitors was not confined to the individuals, but extended often to the parents of the children, for whom work was found or other aid rendered. It was the oldest children's hospital in London. Mr. Lawrence referred to the enormous number of out-patients treated annually, and spoke of hospitals as essentially Christian institutions. He considered the clergy could be most helpful by giving offertories, and in other ways making their needs public, as the late rector of St. John's had done, and to him the hospital owed much. Mr. Hoare spoke of the hospital as standing in what was, without any exception, the most densely-populated district in London. He called attention to the advantages of having a refreshment bar where the out-patients, who had often so long to wait, could obtain what they wanted at a nominal charge. The refreshment bar was so well managed as to be entirely self-supporting. Mr. Eastwood, in proposing that the best thanks should be given to the honorary medical staff, bore witness to the exceeding skill of the surgeons and physicians attached to the hospital. He spoke warmly of the valuable services of the excellent matron, Miss Hunter, and the nurses on the staff, and also of the kind help given by the lady visitors.

The Lord Mayor announced that the secretary had received during the meeting the satisfactory amount of £567 6s. for the funds of the hospital, and the Vicar of Esher promised an offertory. A vote of thanks to the Lord Mayor terminated the meeting.

### THE ROYAL EYE HOSPITAL.—ANNUAL GENERAL MEETING.

The annual general meeting of the Royal Eye Hospital, Southwark, took place on March 25th. There was a small attendance, Professor Malcolm McHardie, vice-president, in the chair. The adoption of the report was moved and carried. The election of Mr. Beerbohm Tree as a vice-president of the hospital was recommended by the council as an acknowledgment of his kindly services, especial thanks being due for his generous gift of the proceeds of a morning performance of "Trilby" at his theatre, whereby some £200 were added to the funds of the institution. In reference to the fitness of patients for admission to the benefits of the hospital, Mr. McHardie remarked that no money was spent at that institution in investigating the position of applicants. They employed no one for this inquisitorial duty, as was the custom elsewhere. The medical officer decided on the ineligibility of cases, and judged if persons were too well dressed to need assistance, or if they were likely to be undesirable associates for other patients, and furnished particulars of such cases to the council after he had dealt with them. The secretary was instructed to convey to the Sister-Matron (Miss Islip) an expression of appreciation from the governors of the institution for the services of herself and the nursing and domestic staffs, much of the comfort and successful working of the hospital being due to the excellent management of this department.

### CITY OF LONDON AND EAST LONDON DISPENSARY. ANNUAL MEETING.

In the absence on account of illness of Mr. F. Sharratt, chairman of the Committee of Management, Mr. W. Cooking presided at the annual meeting, on March 25, of the City of London and East London Dispensary. Mr. Cooking considered it most gratifying to see Mr. F. R. Bluett occupying a seat on the committee, to which he had been unanimously elected on his retirement from the post of secretary, which he had held for a long term of years, and in which his son had succeeded him. A cordial vote of thanks to the medical officers was proposed by the Chairman, seconded by Mr. Bluett, and carried. There had been a great increase in the number of patients and of receipts, which was largely due to the popularity of the medical officers. The latter, in acknowledging the gratifying terms in which their services had been mentioned, alluded to the kind help given by all connected with the institution, and to the willingness of the committee to afford every facility for the treatment of the patients in the matter of drugs, etc.

### THE METROPOLITAN HOSPITAL SATURDAY FUND.

Alderman Sir S. Knill, in the unavoidable absence of the Lord Mayor, presided on Saturday at the Mansion House over the annual meeting in connection with the Hospital Saturday Fund. Mr. Reginald B. D. Acland (the chairman of the fund) stated that their collections in the past year had been fairly satisfactory. He said "fairly satisfactory," because he considered that anything short of £50,000 in a year was not fully satisfactory. They were, however, making a steady increase in their collections. The total receipts in the past year had been £20,354. The street collection showed a decrease of £160, but the shop collections had been £748 more, which was satisfactory, although the amount collected was not a sufficient amount to be contributed by the working classes of the metropolis, considering the benefits which they received from the hospitals of London. The fund made distributions to about 170 different institutions—convalescent homes, hospitals, and dispensaries. The number of letters issued by them in the past year had been 24,562, a large number, but a very small proportion of the total number of patients who went to the hospitals. It might surprise many who were present to hear that one in every four of the population of London annually attended the hospitals, either as an in or as an out patient—that was 1,100,000 persons went to the metropolitan hospitals every year. Cardinal Vaughan proposed the first resolution, expressing approval "of the principle of soliciting small regular contributions in aid of the metropolitan medical charities from those employed in the workshops and places of business of London." Canon Shuttleworth seconded the motion, which was carried unanimously. Colonel Sir Herbert C. Perrott proposed a resolution approving the efforts of the fund "to spread the knowledge of ambulance work among those employed in the workshops and places of business in London, to train them to render first aid in case of accident or sudden illness, and also to provide them with the necessary appliances for this purpose." General Sir George H. S. Willis afterwards presented first-aid certificates of the St. John Ambulance Association to the students of the Hospital Saturday Fund.

